# Supplementary material for: Cell surface crowding is a tunable energetic barrier to cell-cell fusion
Source: Nat Commun. 2025 Aug 4;16:7158. doi: 10.1038/s41467-025-62330-8 (PMC12322221; doi:10.1038/s41467-025-62330-8)
Supplement: Supplementary file 1 — Supplementary Information [file 41467_2025_62330_MOESM1_ESM.pdf]

Supplementary Information

"Cell surface crowding is a tunable energetic barrier to cell-cell fusion"

Daniel S.W. Lee*, Liya F. Oster*, Sungmin Son, and Daniel A. Fletcher

Supplementary Note

Estimation of apposition energy

We developed a scaling argument to describe the relationship between surface crowding and fusion. It has previously been shown that the energy penalty for insertion of a molecule into a polymer brush, here defined as ΔU_{s}, can be defined as the product of the osmotic pressure of the brush layer, $\Pi(\phi)$, and the effective volume of the inserted molecule, V_{ab}, which is an antibody molecule in the case of our sensor. To convert this measured crowding energy to that required to clear a surface of some arbitrary size, e.g., as would be required for fusion, we simply rescale this energy by the relative volume of the fusion site compared to that of the antibody, i.e., $\Delta U_{\mathrm{patch}} = \Delta U_{\mathrm{s}} L^2{}_{\mathrm{fuse}}/L^2{}_{\mathrm{ab}}$. We estimate that the size of the antibody is $L_{\mathrm{ab}} \approx 10-15$nm, while we estimate the size of the fusion patch on one membrane to be $L_{\mathrm{fuse}} \approx$ 50-100nm based upon imaging done in the *Drosophila* myoblast[1]. The probability that this occurs at equilibrium will go as the exponential of this energy, according to a standard Boltzmann distribution, i.e., $p = \exp\left(-\Delta U_{\mathrm{patch}}/k_{\mathrm{B}}T\right)$. Assuming that this must occur independently on two apposed surfaces, the probability will scale additively with the crowding energy, and so the energy required for close apposition of two membranes is $\Delta U_{\mathrm{app}} = 2\Delta U_{\mathrm{s}} L^2{}_{\mathrm{fuse}}/L^2{}_{\mathrm{ab}}$.

Supplementary Figures

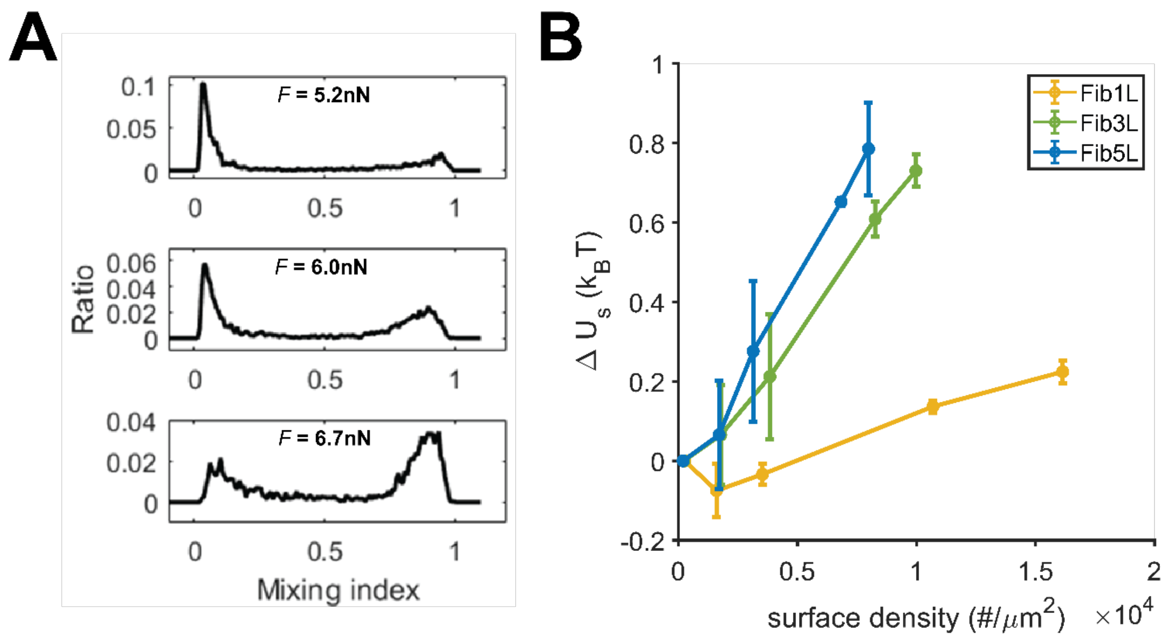

Supplementary Fig. 1: Computation of mixing ratio and crowding interpolation. **A**, The mixing ratio was computed by taking the percentage of beads found in the peak in the histogram corresponding to a higher fluorescence ratio. **B**, The ΔU_s for Fibcon repeats on beads was computed by using previously published data[2] (blue, yellow, green) and linearly interpolating for the surface densities used in the centrifugation experiments.

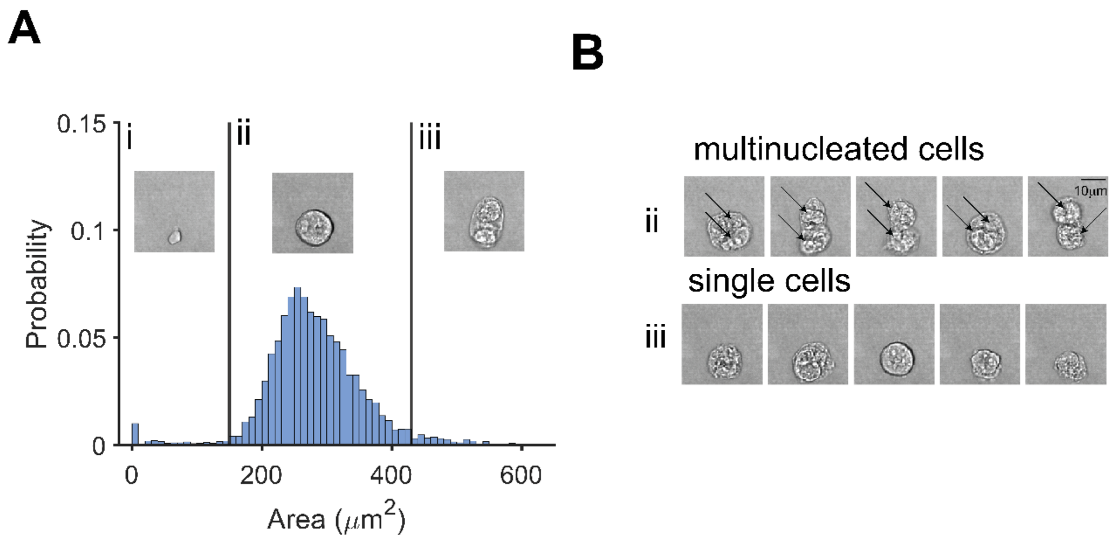

Supplementary Fig. 2: Identifying fused cells by imaging flow cytometry. **A,** High throughput imaging flow cytometry was used to determine the fusion percentage in the hybridoma experiments. Bright field images (*inset*) were taken for each event and segmented, producing a histogram of cell areas. A roughly 2-standard-deviation-threshold was used to separate i) debris, ii) single cells, and iii) multinucleated hybridomas. **B,** This thresholding approach was validated by visual inspection. Five example images are shown for each condition. The events categorized as iii) multinucleated hybridomas all showed multiple nuclei, while the ii) single cells did not but did appear to be intact single cells.

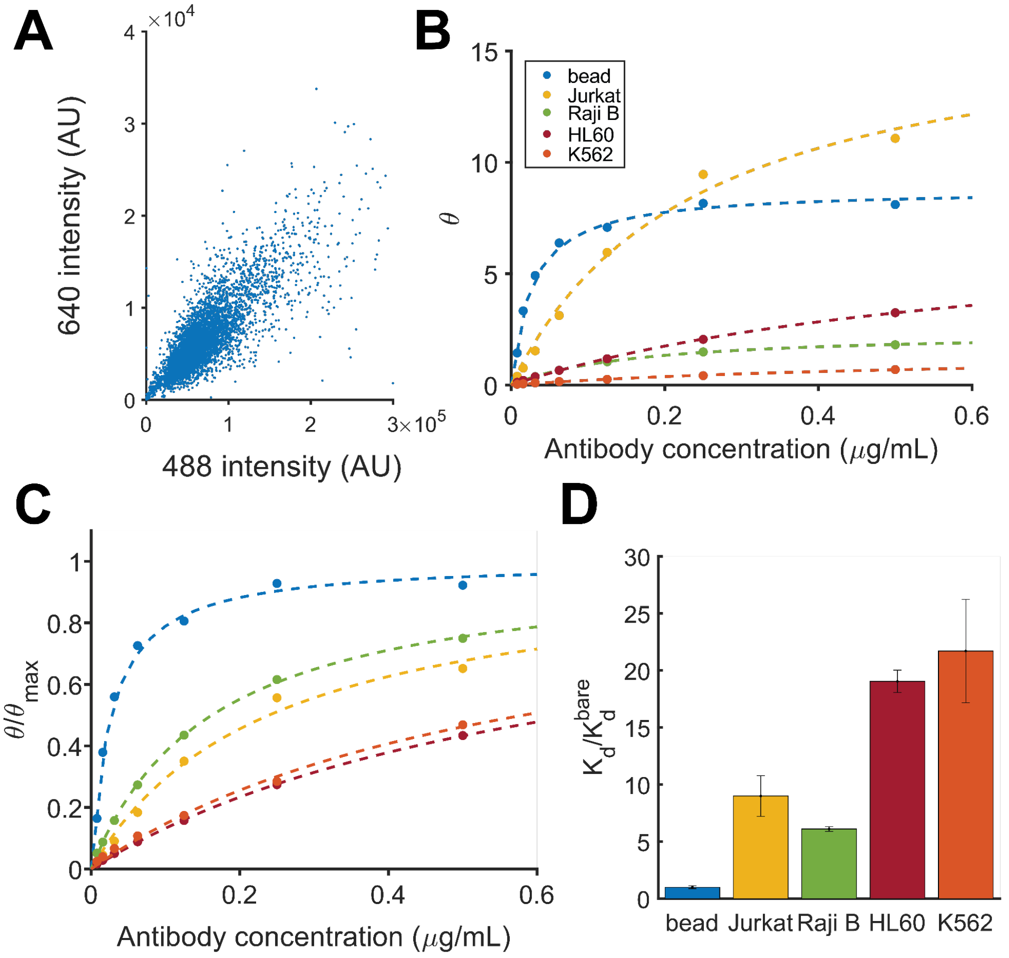

Supplementary Fig. 3: Crowding probe analysis. A, Example flow cytometry data for the crowding probe. For each antibody/cell condition, the crowding probe occupancy ratio θ was calculated by taking the ratio of antibody (640 fluorescence) to oligo (488) for each cell. A linear trend suggests that sensor-oligo binding does not affect crowding readouts, and there is a constant binding ratio θ within this sample. **B,** Occupancy ratio was calculated for a range of antibody concentrations for each cell condition since saturation ratios may differ between cell types. Fluorescent ratios were fit to the Langmuir isotherm, i.e., $\theta = \theta_{\mathrm{max}} \frac{c}{c+K_{\mathrm{d}}}$ (dashed line). **C,** The occupancy ratio, normalized θ_{max}, i.e., maximum sensor occupancy, across cell types, which we treated as a free parameter, was plotted for each condition for visual comparison of the K_{d}. The K_{d}, was calculated to assess the affinity change due to cell surface crowding and normalized to that of a bead (K_{d}^{0}), giving the calculated crowding energy barrier. **D**, K_{d} values for a range of suspension cell types (n>10000 cells per condition).

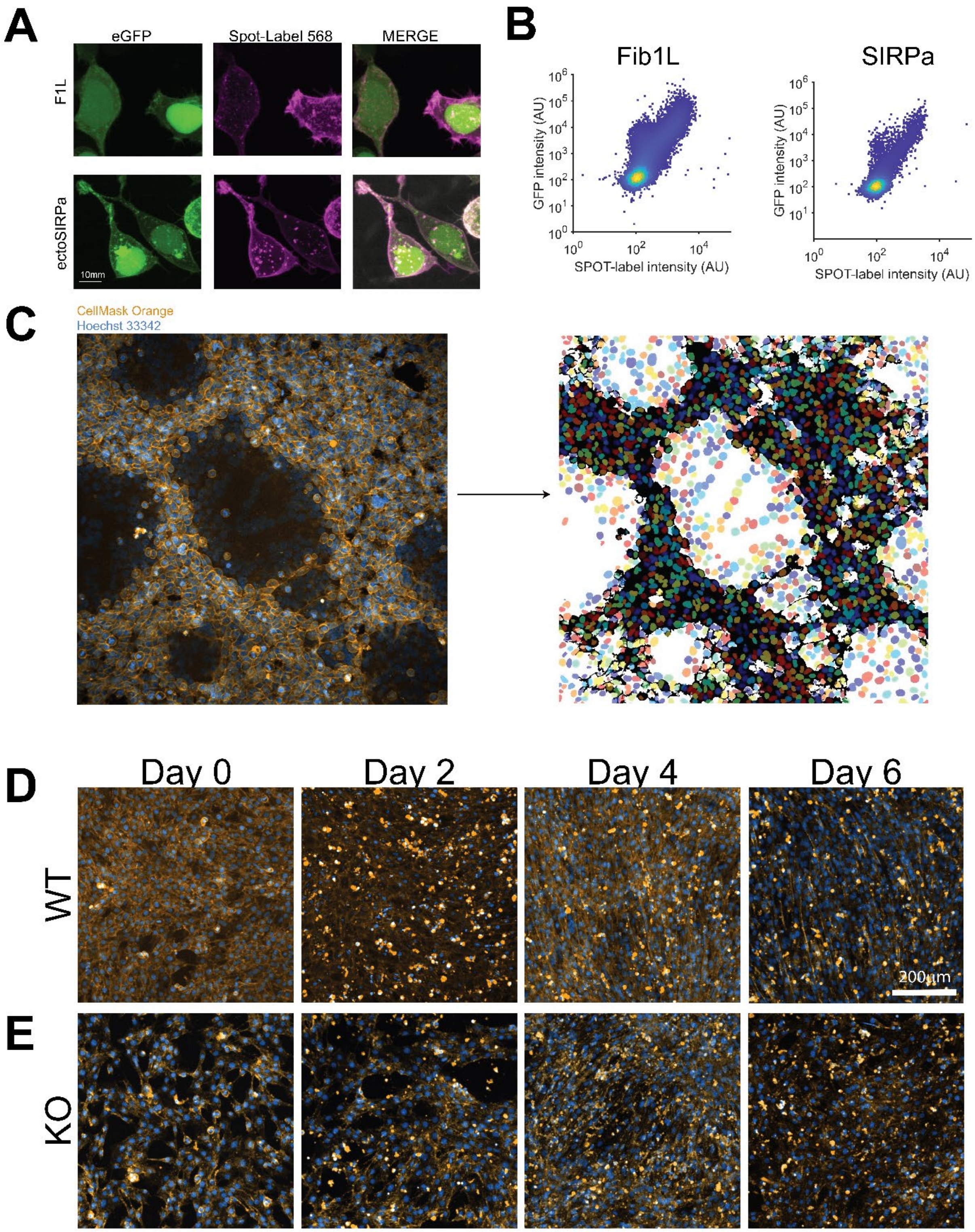

Supplementary Fig. 4: Validation of cell-cell fusion assays. A, Transfected cells were stained with SPOT-label 568 and imaged or quantified by flow cytometry. Surface localization of SPOT-label and nuclear localization of fluorescent protein were observed.

B, A direct proportionality between GFP and spot-label stain intensity were observed by flow cytometry. **C,** For p14 experiments, cells were stained with CellMask Orange and Hoechst 33342 to independently image the cell membrane and nucleus (left). Example segmentation of syncytia (black/white) and nuclei (right). Nuclei overlapping the white region were defined as in syncytia and those overlapping the black region were defined as not in syncytia. **D,** Microscopy for validation of myoblast fusion. Following serum starvation, wild type C2C12 cells were stained with CellMask Orange and Hoechst 33342, demonstrating myotube formation by day 4. **E,** Myomaker knockout cells did not fuse but showed slight elongation following identical starvation treatment.

References

1. Sens, K. L. et al. An invasive podosome-like structure promotes fusion pore formation during myoblast fusion. *J Cell Biol* **191**, 1013–1027 (2010).
2. Takatori, S. C., Son, S., Lee, D. S. W. & Fletcher, D. A. Engineered molecular sensors for quantifying cell surface crowding. *Proceedings of the National Academy of Sciences* **120**, e2219778120 (2023).
