# Supplementary material for: Cell surface crowding is a tunable energetic barrier to cell-cell fusion
Source: Nat Commun. 2025 Aug 4;16:7158. doi: 10.1038/s41467-025-62330-8 (PMC12322221; doi:10.1038/s41467-025-62330-8)
Supplement: Supplementary file 2 — Reporting Summary [file 41467_2025_62330_MOESM2_ESM.pdf]

nature portfolio

Corresponding author(s): Daniel A. Fletcher

Last updated by author(s): 06/27/2025

Reporting Summary

Nature Portfolio wishes to improve the reproducibility of the work that we publish. This form provides structure for consistency and transparency in reporting. For further information on Nature Portfolio policies, see our Editorial Policies and the Editorial Policy Checklist.

Statistics

For all statistical analyses, confirm that the following items are present in the figure legend, table legend, main text, or Methods section.

n/a	Confirmed	
☐	☒	The exact sample size (*n*) for each experimental group/condition, given as a discrete number and unit of measurement
☐	☒	A statement on whether measurements were taken from distinct samples or whether the same sample was measured repeatedly
☐	☒	The statistical test(s) used AND whether they are one- or two-sided<br>*Only common tests should be described solely by name; describe more complex techniques in the Methods section.*
☐	☒	A description of all covariates tested
☐	☒	A description of any assumptions or corrections, such as tests of normality and adjustment for multiple comparisons
☐	☒	A full description of the statistical parameters including central tendency (e.g. means) or other basic estimates (e.g. regression coefficient) AND variation (e.g. standard deviation) or associated estimates of uncertainty (e.g. confidence intervals)
☐	☒	For null hypothesis testing, the test statistic (e.g. *F*, *t*, *r*) with confidence intervals, effect sizes, degrees of freedom and *P* value noted<br>*Give P values as exact values whenever suitable.*
☒	☐	For Bayesian analysis, information on the choice of priors and Markov chain Monte Carlo settings
☒	☐	For hierarchical and complex designs, identification of the appropriate level for tests and full reporting of outcomes
☒	☐	Estimates of effect sizes (e.g. Cohen's *d*, Pearson's *r*), indicating how they were calculated

Our web collection on statistics for biologists contains articles on many of the points above.

Software and code

Policy information about availability of computer code

Data collection	Nikon Elements was used to collect data by microscopy; Attune Cytometric Software (Thermo) was used to collect flow cytometry data.
Data analysis	MATLAB and ImageJ were used to analyze data. MATLAB was used to analyze flow cytometry data. Custom routines are publicly available at

For manuscripts utilizing custom algorithms or software that are central to the research but not yet described in published literature, software must be made available to editors and reviewers. We strongly encourage code deposition in a community repository (e.g. GitHub). See the Nature Portfolio guidelines for submitting code & software for further information.

Data

Policy information about availability of data

All manuscripts must include a data availability statement. This statement should provide the following information, where applicable:

- Accession codes, unique identifiers, or web links for publicly available datasets
- A description of any restrictions on data availability
- For clinical datasets or third party data, please ensure that the statement adheres to our policy

All data associated with this manuscript are available at the Center for Open Science, at DOI 10.17605/OSF.IO/AF2Q7, and any further requests may be directed to the corresponding author.

Research involving human participants, their data, or biological material

Policy information about studies with human participants or human data. See also policy information about sex, gender (identity/presentation), and sexual orientation and race, ethnicity and racism.

Reporting on sex and gender	No human data or participants were used in this study.
Reporting on race, ethnicity, or other socially relevant groupings	No human data or participants were used in this study.
Population characteristics	No human data or participants were used in this study.
Recruitment	No human data or participants were used in this study.
Ethics oversight	No human data or participants were used in this study.

Note that full information on the approval of the study protocol must also be provided in the manuscript.

Field-specific reporting

Please select the one below that is the best fit for your research. If you are not sure, read the appropriate sections before making your selection.

☒ Life sciences ☐ Behavioural & social sciences ☐ Ecological, evolutionary & environmental sciences

For a reference copy of the document with all sections, see nature.com/documents/nr-reporting-summary-flat.pdf

Life sciences study design

All studies must disclose on these points even when the disclosure is negative.

Sample size	Sample size was not predetermined.
Data exclusions	No data were excluded from experiments in this study.
Replication	All experiments were performed in at least 3 biologically independent replicates and the averaged results are shown as the final data.
Randomization	Randomization was not relevant to this study as all analysis was unbiased and quantitative.
Blinding	Blinding was not relevant to this study as all analysis was unbiased and quantitative.

Reporting for specific materials, systems and methods

We require information from authors about some types of materials, experimental systems and methods used in many studies. Here, indicate whether each material, system or method listed is relevant to your study. If you are not sure if a list item applies to your research, read the appropriate section before selecting a response.

Materials & experimental systems

n/a	Involved in the study	
☐	☒	Antibodies
☐	☒	Eukaryotic cell lines
☒	☐	Palaeontology and archaeology
☒	☐	Animals and other organisms
☒	☐	Clinical data
☒	☐	Dual use research of concern
☒	☐	Plants

Methods

n/a	Involved in the study	
☒	☐	ChIP-seq
☐	☒	Flow cytometry
☒	☐	MRI-based neuroimaging

Antibodies

Antibodies used	Alexa Fluor 647-conjugated anti-biotin antibody BK-1/39 (Santa Cruz Biotechnology, sc-53179)
Validation	This antibody was previously validated in its use by our lab in Joffe et al 2020 Nat Methods; Takatori 2023 PNAS; and others. Its binding is directly related to the integration of the biotin oligo as seen in S2A.

Eukaryotic cell lines

Policy information about cell lines and Sex and Gender in Research

Cell line source(s)	Cell lines were obtained from the cell culture repository at Barker Hall at UC Berkeley, or from Pengpeng Bi (University of Georgia), and include human and murine cell lines.
Authentication	Cell lines were not authenticated.
Mycoplasma contamination	All cell lines tested negative for mycoplasma contamination.
Commonly misidentified lines (See ICLAC register)	N/A

Plants

Seed stocks	N/A
Novel plant genotypes	N/A
Authentication	N/A

Flow Cytometry

Plots

Confirm that:

☒ The axis labels state the marker and fluorochrome used (e.g. CD4-FITC).

☒ The axis scales are clearly visible. Include numbers along axes only for bottom left plot of group (a 'group' is an analysis of identical markers).

☒ All plots are contour plots with outliers or pseudocolor plots.

☒ A numerical value for number of cells or percentage (with statistics) is provided.

Methodology

Sample preparation	Cell samples were cultured and directly harvested from liquid culture, using EDTA to dissociate if necessary.
Instrument	Attune CytPix Flow Cytometer (Thermo Fisher A51849)
Software	Attune Cytometric Software was used to acquire the data, and custom MATLAB code, available at at DOI 10.17605/OSF.IO/AF2Q7, was used to analyze it.
Cell population abundance	All cell populations were uniform and gated only for live cells and singlet/doublet discrimination.
Gating strategy	Cells were gated for live cell populations and evaluated based upon imaging and staining data (Fig S1A, Fig S2).

☒ Tick this box to confirm that a figure exemplifying the gating strategy is provided in the Supplementary Information.
